# Cases of Poisoning with Arsenic

**Published:** 1851-07

**Authors:** Samuel Thompson

**Affiliations:** Albion, Edwards Co., Ill’s., President of the State Medical Society, etc.


THE NORTH-WESTERN
MEDICAL AND SURGICAL JOURNAL.

VOL. IV.] JULY, 1851. [No. 2.

### Part I.—Original Communications.

ARTICLE I.

*Cases of Poisoning with Arsenic.*—By SAMUEL THOMPSON, M.D., of Albion, Edwards Co., Ill's., President of the State Medical Society, etc.

Cases of Arsenical Poisoning are among the rare incidents in the experience of a physician residing in the country. To carefully communicate to others the records of any such cases, as occur to any of us, I consider therefore to be a special duty. There are also other reasons why in reference to such accidents especially, each should publish his experience and ideas, inasmuch as our highest authorities only form *their* opinions from the gleanings of settled records. And there are *yet*, some points, regarding both the action of the poison and the means of remedy, upon which considerable obscurity still exists.

The following extract from Dr. Christisons work on Poisons, (an allowed standard authority,) comprises I believe, the generally seconded views upon the subject. He classes the effects of Arsenic in three divisions. "In the first order of cases "Arsenic produces symptoms of irritation or inflammation "along the course of the alimentary canal; such cases are the "most frequent of all. The person commonly survives 24 "hours, seldom more than three days, but instances of the "kind have sometimes proved fatal in a few hours, and others

"lasted for weeks." "In regard to the ordinary progress of "the symptoms, the first of a decisive character, are sickness "and faintness," sometimes occurring in a few moments, seldom delayed more than an hour.

"The second variety of poisoning with Arsenic includes a few cases, in which the signs of inflammation are far from violent, or were altogether wanting, and in which death ensues in five or six hours, or a little more, at a period too early for inflammation to be properly developed." The symptoms, Dr. C. thinks are then to be refered, to what he considers the probable cause of death in most cases. "An action on some remote organ." "The symptoms in these cases occasionally amount to absolute narcotism, and succeeding to vomiting, extreme faintness, at times amounting to deliquium." This variety has only been observed when the dose has been *very large*, or in a *state of solution*, or, (as stated) when the Arsenic was in little masses.

"The third variety of poisoning with arsenic, places in a clear view its occasional action on the nervous system. This occurs chiefly in persons, who from having taken but a small quantity, or from having vomiting soon after, are eventually rescued from destruction. But it has also been met with in some cases where death ensued after a protracted illness; in such cases the progress of poisoning may be divided into two stages. The first train of symptoms is exactly that of the first or inflammatory variety, and is commonly developed in a very perfect and violent form. In the second stage the symptoms are referable to nervous irritation. These generally come on when the former begins to subside; yet sometimes they make their appearance clearer, while at the same time the signs of inflammation in the alimetary canal continue violent. The nervous affection varies in different individuals; the most formidable is coma; the slightest, a peculiar imperfect palsy of the arms or legs, resembling what is occasioned by lead, and between these extremes have been observed epileptic fits, or an affection resembling hysteria or mania."

Now the cases with the details of which I am about to present you, undoubtedly belong to the third or last class, at the same time the symptoms deviated somewhat from Dr. C.'s description, and it was probable, indeed, that in the first instance the amount of poison taken was large, as it will be seen by the analysis of Dr. David D. Owen, that in the contents of the vial I sent him containing the matters left at the bottom of the vessel in which the separated food had been cooked, there was "a considerable quantity of arseniuos acid." Now it was evident the arsenic must have been introduced during or before the process of boiling, and as boiling water dissolves about 77.75 of arsenic to 1000 parts of the water, the proportion of arsenic contained in the food was most probably considerable.

It was about dark on the 9th of July last, that I was summoned in a great hurry to visit a family four or five miles distant, who were supposed to be poisoned. I enquired of the messenger, in what way it was supposed the accident had occurred, and what was the poison suspected to have been taken. He informed me that they had been using arsenic to poison flies, and they believed they must have gotten some in their food, as the whole family, eight in number, were taken bad either directly or shortly after dinner. I therefore took with me such antidotes as the suspicion suggested.

Now as in these matters, details are often of great importance to the correct appreciation of facts, I will run the risk of being tedious, by describing the suffering parties. There was one young woman, of about 25 years of age, whom I shall designate as No. 1, stout, plethoric, of sanguine temperament, fair florid complexion, and black hair. (Her mother died of Phthisis.) Another of about the same age, No. 2, rather slight and of nervous sanguine temperament and fair hair and complexion. One man, No. 3, aged about 55, of robust make and sanguine bilious temperament, has been a hard drinker all his life, but rarely gets drunk. No. 4, a man about 50, of the same general description and habits. No. 5, a youth about 19 of robust healthy frame, and tolerably temperate habits. No. 6, a man of about 25, very fair complexion and sandy hair, and also a soaker. No. 7, a young man of about 21, tall, stout, muscular, of sanguine bilious temperament, and also following the custom of the establishment, of taking fermented liquors habitually and freely, and No. 8, a lad of about 16, a brother to the last.

It appears that immediately after dinner, which consisted of Windsor beans and new potatoes boiled together with some bacon in an iron pot, besides rice, butter, bread, etc., the two young women were first attacked with vomiting, but without suspecting what was the cause, while No. 3, the master of the house, whose robust and rugged constitution seemed to withstand the influence of the poison, walked off to his work in the harvest field, but after going a few yards, he also began to vomit; and when he had proceeded about 200 yards, sank down faint and exhausted, where he was shortly after found by the two young women, who, temporarily recovering from their first attack of vomiting, went after him and assisted to get him into the house. At this time, 1 o'clock P, M., all the family were suffering more or less from what they now felt sure must be poison, but the master of the house still obstinately persisted it could not be from arsenic.* At this time No. 4 becoming alarmed, declared that if he was to die poisoned, he would die drunk, and at once drank off about a pint of whiskey. It does not appear that any thing more was done till about five or six o'clock, (meanwhile the parties had been getting worse,) when the lad, No. 8, being the least effec-

*He had so often been blamed for the careless way in which he kept arsenic about the house, that being a very self-willed, pertinacious man, he would by no means allow that he had been to blame.

ted, managed to crawl across the fields to the adjoining farm to obtain help, whereupon a messenger was dispatched for the uncle of No. 2, a practitioner of medicine residing about five miles off, and another person came for me.

When I arrived at the house I found the two young women in violent convulsions; the hands clenched, the feet extended by the spasmodic contraction of the gastrocnemeii, etc. The jaws locked, and at intervals well marked opisthotonos, so that in fact they stood on their heels and their heads. These two as well as the others, complained of harsh metalic taste, burning at the epigastrium, some had head-ache, and some diarrhœa. One of the young men, (No. 5,) suffered also from tetanic spasms. The pulse in all the cases was weak and irregular, and not frequent, and the skin cool.

I at once administered to all who would take it, as much Percipitated Carbonate of Iron as they could swallow. To some I gave it in molasses, to others, who objected to the molasses, it was given, just made into thin paste with water; the doses being repeated as fast as they were vomited. All who took the iron, felt almost immediate relief from the excessive burning, aching and sinking, from which they had previously suffered. But two of those who were the worst, and who subsequently remained the worst, namely: the young man No. 5, and the young woman No. 2, for more than an hour obstinately refused to take any thing, but at last her sufferings, added to the evident relief obtained by others, induced No. 2 to submit, though she would not take the Iron. I therefore gave Calcined Magnesia in teaspoon-full doses, which almost immediately mitigated her sufferings. But it appeared to me that the relief was neither so complete nor so *permanent* as that obtained from the Iron. No. 5 took the Iron, but sparingly.

Now it will be remembered that after the full repeated doses of Carb. Iron, or of the Magnesia, all of the patients appeared relieved of their most distressing symptoms, and they all passed a tolerably good night. But the next morning early, (the 10th,) the tetanic spasms in the young woman recurred, as also in the youth, No. 5, but all appeared easy in the intervals except No. 2, who complained of a feeling of weight on the vertex. The symptoms of nervous irritation now predominated in each case. It was remarkable that whiskey to those persons so well used to it, did not appear to agravate the symptoms, which if irritation or inflammation of the mucus membrane of the stomach had existed, we should suppose must have been the case. Three of the men, who appeared to suffer but little to-day, insisted on going into the harvest field, and as they afterwards told me, while *drinking* and *sweating freely*, they felt almost well; but as soon as the sweating ceased, all their bad symptoms returned. During the fits of convulsions, which with frightful intensity would sometimes last for an hour, consciousness was never lost; though the power of speech was nearly gone, the voice never rising above a hissing whisper. The desire for cold drinks during the continuance of the spasms was intense. In the case of No. 5, it was as much as four stout men could do during the fits to prevent him beating himself to pieces against the floor or walls; and throughout their sickness, after the first night, constipation, *not* diarrhœa, had continually to be obviated. I continued in attendance regularly until the 16th.

I have omitted to state that on the night of the 10th, the two young women were troubled for a time with excessive pruritis over the whole body, with occasional short flashes of fever.—On No. 2, an eruption, almost resembling impetigo, came out over the legs, but this might have originated from the desperate frictions to which they had been (according to popular usage,) subjected during the spasms before I arrived. On the second night, during a long continued epileptic fit, with hard full pulse at both radials, and carotids, with aphonia and heat of scalp, I drew *about* ℥xx blood from No. 5. I could only guess at the quantity, as I had to perform the operation during the most violent struggling and resistance, though he was held by three men. He became calm afterwards, but it did not appear to decrease the frequency of the paroxysms. The least exertion, the smallest excitement of mind or body, being for sometime succeded by these fits, though by degrees getting milder each time, and these attacks were most apt to occur towards night.

Now the course I pursued was as follows: After the first twelve hours I concluded it was useless to administer antidotes, the power of which must depend upon their coming in contact with the poison in the stomach, and forming insoluble compounds with it. The object next to be sought, I considered must be to remedy the evils already produced, and to remove such portions of the poison as remained, not in the stomach or bowels, but as I believe, absorbed into the blood and thereby deposited in the tissues.

For this purpose I employed occasional doses of Calomel combined with Hyosciamus, with Spts. Nitre and Compound Powder of Jalap, and each night Morphia with Quinine, to soothe and sustain the nervous system, and after the first three or four days I put them all upon a steady course of the Tinct. Ferri Mur. gtt. xx ter. die.

Now writers on Toxicology appear chiefly to have directed their attention to the treatment of cases of poisoning by arsenic in *powder*. Their instructions are chiefly in regard to the gastric or arteric irritations. Dr. Christoson says, after the poison has been removed from the stomach, "Two indications of cure remain to be fulfilled, viz: to allay the inflammation of the alimentary canal, and to support the system under that extraordinary depressiom which it undergoes in the generality of cases. *Were it not for the latter* of these objects, the treatment would be both obvious and generally successul."—[Christoson: page 335: 3d London edition.]

I quote from Christoson alone, because he appears to be the standard and basis of most others. Dr. Pareira, in his valuable and copious work on Materia Medica, gives but a synopsis of Dr. C.'s facts and opinions. In Dr. Apjohn's article upon toxicology in the Cyclopedia of Practical Medicine, he refers briefly, under the head of Arsenic, to its effects upon the nervous system, but it is merely to mention the fact. He denies the absorption of arsenic into the blood as its *modus operandi*, though he rather favors the views of Morgan and Addison, which refer the effects to the action of the poison upon the nervous fibrils spread over the lining membrane of the vascular system, (apparently overlooking the fact that this is merely a *mode* of action *through absorption*.) And thus it would act directly on the sentient extremeties of the ganglionic nerves, for it is these which supply the blood-vessels, and thence producing either irritation of the ganglionic system and its dependencies, or prostration, just in proportion to the concentration of the cause in relation to the resisting power of the individual. But if poison is thus taken into the blood, what becomes of it? Is it all excreted at once by the alvine discharges, etc., or is it only part exrceted and part deposited? If we admit the latter, which we can hardly doubt, from the analogy of Ferruginous and other medicinal metalic substances, then what are the effect of these deposited portions? are they inert, or do they combine to exert their usual irritating effects?*

Now it appears to me that not only are these important to a full understanding of some of the phenomena I have recorded, but also as a guide to correct and scientific treatment, or in other words, to acting in reference to the real condition of

---

*Any substance absorbed from the alimentary canal, (by the veins,) goes by them to the liver, thence to the right side of the heart, thence through the lungs, thence to the left side of the heart, and thence over the system. Its first effects then would be upon the ganglionic system, as exposed to its action in the veins, liver, heart, etc. Its second effects would be again upon the heart, and then upon the general organism; and hence would result poisoned nutrition, which could only be removed by the absorbent action of the veins or lymphatics, and thence excreted with other morbid or effete elements.

the system and the cause if its suffering. If morbid matters are in the blood or in the tissues, the excreting organs should remove them.

The object in correct practice should be first so understand, if possible, where and how nature suffers; and where she fails to help herself, we should aid her weakness; and when she injures herself by her efforts, we should control her excesses. If we were treating an injury of the surface of the body, (as from a splinter or a thorn,) we would both strive to moderate the irritation, to remove the cause, and help nature to repair the evil done; and one effort would contribute to the success of others. If we find a person then suffering from the nervous irritation of arsenical poisoning, is it sufficient to sooth the nervous irritation, or must we not rather set all the outlets of the body to work, to remove the cause remaining? If my views are correct, that such cause is really left in the tissues of the body, then that the latter is a prominent duty cannot be doubted.

In this place I mention an idea that has occurred to me, which, though merely a hypothesis, may suggest to others further enquiry. The known tendency of Arsenic to combine with Iron is the basis of all our effectual antidotes to its effects. May not the combination of the arsenic with the iron of the blood account for some of its effects?

It will also be remembered that I mentioned that whiskey taken in considerable quantities, did not, (to say the least of it,) appear to produce any injurious effects. Dr. Christoson, at page 337, says, when speaking of the practice proper in the advanced stages of poisoning with arsenic, "That the principle object is to support the system by mild nourishment, avoiding at the same time stimulant diet of every kind, *but especially spiritous and vinous liquors*."

I before remarked that had gastric irritation been the prominent symptom, the propriety of this was evident; but I think it is further evidence that the stomach bore but little share in the injury, and I suspect the *accustomed stimulus*, only aided nature to sustain its own action and carry on the elimination of the poison.

For myself I confess, reasoning only upon the recorded observatinos of others, I by no means feel sure that inflammation of the grstric surface is essential to the most violent effects of the arsenic, because in those cases in which death has occurred within two days, it has by no means been found as a constant circumstance.

Arsenic is not a corrrosive poison. The after effect of inflammation of the stomach may be a secondary result fróm the poisoned condition of the system, in common with inflammation and gangrene of other parts, etc. etc. The most soluble preparations of arsenic are the most virulent; and our very antidotes act on the principle of forming an insoluble compound, perhaps therefore we have too readily supposed that gastric inflammation is a prominent and direct cause of death.

I submit these views and suggestions with great humility, for my experience herein, is small; but I conceive we should never let respect for authorities, stay us from enquiry.

P. S. I have to acknowledge my obligations to that eminent Chemist and Geologist, Dr. David D. Owen, of New Harmony, for his polite and able analysis of the suspected articles. His report I would copy but for the already to great length of this article.

*April* 30, 1851.

